# ΔNp63 Regulates Radioresistance in Human Head and Neck Squamous Carcinoma Cells

**DOI:** 10.3390/cimb45080394

**Published:** 2023-07-27

**Authors:** Kota Sato, Hironori Yoshino, Yoshiaki Sato, Manabu Nakano, Eichi Tsuruga

**Affiliations:** 1 Department of Radiation Science, Graduate School of Health Sciences, Hirosaki University, Hirosaki 036-8564, Aomori, Japanysatoo@hirosaki-u.ac.jp (Y.S.); tsuru@hirosaki-u.ac.jp (E.T.); 2 Department of Bioscience and Laboratory Medicine, Hirosaki University Graduate School of Health Sciences, Hirosaki 036-8564, Aomori, Japan; mnakano@hirosaki-u.ac.jp

**Keywords:** ΔNp63, radioresistance, head and neck squamous cell carcinoma, ionizing radiation, karyopherin-β1, karyopherin-α, radiation-induced apoptosis

## Abstract

Radiation therapy is commonly used to treat head and neck squamous cell carcinoma (HNSCC); however, recurrence results from the development of radioresistant cancer cells. Therefore, it is necessary to identify the underlying mechanisms of radioresistance in HNSCC. Previously, we showed that the inhibition of karyopherin-β1 (KPNB1), a factor in the nuclear transport system, enhances radiation-induced cytotoxicity, specifically in HNSCC cells, and decreases the localization of SCC-specific transcription factor ΔNp63. This suggests that ΔNp63 may be a KPNB1-carrying nucleoprotein that regulates radioresistance in HNSCC. Here, we determined whether ΔNp63 is involved in the radioresistance of HNSCC cells. Cell survival was measured by a colony formation assay. Apoptosis was assessed by annexin V staining and cleaved caspase-3 expression. The results indicate that ΔNp63 knockdown decreased the survival of irradiated HNSCC cells, increased radiation-induced annexin V^+^ cells, and cleaved caspase-3 expression. These results show that ΔNp63 is involved in the radioresistance of HNSCC cells. We further investigated which specific karyopherin-α (KPNA) molecules, partners of KPNB1 for nuclear transport, are involved in nuclear ΔNp63 expression. The analysis of nuclear ΔNp63 protein expression suggests that KPNA1 is involved in nuclear ΔNp63 expression. Taken together, our results suggest that ΔNp63 is a KPNB1-carrying nucleoprotein that regulates radioresistance in HNSCC.

## 1. Introduction

Head and neck squamous cell carcinoma (HNSCC) develops in the oral cavity and pharynx. Since this organ is associated with eating, breathing, and speaking, HNSCC has a significant impact on patient quality of life. Although some progress has been made in recent years in treatment, such as targeted molecular therapy, the prognosis for HNSCC patients remains poor [1,2].

Radiation therapy is commonly used for treating HNSCC. Although it has the advantage of preserving tissue function and morphology, the development of radioresistant cells is an obstacle to the overall efficacy of radiation therapy. Since the emergence of radioresistant cancer cells is a risk for cancer recurrence [3], it is necessary to understand the molecular basis of radioresistance to improve treatment for HNSCC [4,5].

The karyopherin-α (KPNA) and karyopherin-β1 (KPNB1) families are involved in the nuclear transport system and are responsible for transporting nuclear proteins via nuclear localization signals (NLSs) into the nucleus. KPNA recognizes NLSs and binds to nucleoproteins, whereas KPNB1 binds to the KPNA-nucleoprotein complex and functions as a transport carrier. Recent evidence has highlighted the importance of cancer-specific alterations in KPNA subtypes in cancer biology, as well as the benefits of targeting KPNB1 for cancer therapy [6,7,8,9]. We previously reported that KPNB1 inhibition increases cytotoxicity in various cancer cells, including HNSCC cells. It also increases radiosensitivity and enhances radiation-induced apoptosis specifically in HNSCC cells [10], suggesting that KPNB1 can transport nucleoproteins that specifically regulate radioresistance in HNSCC cells.

ΔNp63 is an isoform of the tumor protein p63 (TP63), which is a member of the TP53 family. ΔNp63 lacks an N-terminal trans-activation domain, which is present in the other isoform, TAp63 [11]. C-terminal alternative splicing generates additional isoforms, including α, β, and γ in TP63 [12]. Similarly to TP53, TAp63 is known to function as a tumor suppressor gene by inducing cell cycle arrest and apoptosis through transactivation of its target genes [13]. On the contrary, ΔNp63 serves as an oncogene, and ΔNp63 isoforms, particularly ΔNp63α, are overexpressed in SCC [14,15,16,17]. For example, ΔNp63 is known to trigger a variety of survival signaling cascades such as epithelial growth factor receptors [18]. In addition, ΔNp63 is reported to promote the proliferation of cancer cells by repressing the transcription of cell cycle regulators such as p21, and to inhibit apoptosis in SCC by suppressing the expression of pro-apoptotic factors such as Noxa [19,20]. Furthermore, there are some reports suggesting the importance of ΔNp63 in DNA damage responses. Bretz et al. reported that ΔNp63 activates the Fanconi anemia DNA repair pathway and limits the efficacy of DNA-damaging agent cisplatin treatment in SCC [21]. Recently, Kudo et al. also reported that ΔNp63α represses the p53-related DNA damage response after irradiation [22].

In a previous study, we demonstrated a radiosensitizing effect of KPNB1 inhibition in ΔNp63-expressing cell lines. KPNB1 inhibition caused the diffusion or reduced nuclear localization of ΔNp63 in HNSCC cells [10]. Based on these results, we hypothesized that ΔNp63 may be a KPNB1-carrying nucleoprotein that regulates the radioresistance of HNSCC cells; however, it is unclear whether ΔNp63 is significantly involved. In the present study, our findings suggest that ΔNp63 is a KPNB1-carrying nucleoprotein which regulates radioresistance in HNSCC.

## 2. Materials and Methods

### 2.1. Reagents

PBS(−) (Ca^2+^, Mg^2+^-free Dulbecco’s phosphate buffered saline) and mouse anti-α-tubulin antibody (cat. no. 017-25031) were purchased from Wako Pure Chemical Industries, Ltd. (Osaka, Japan). Propidium iodide (PI) was purchased from Sigma-Aldrich (St. Louis, MO, USA). Annexin V Binding Buffer, FITC-labeled annexin V (FITC-annexin V), and rabbit anti-p63(ΔN) antibody (#619001) were purchased from BioLegend (San Diego, CA, USA). Rabbit anti-cleaved caspase-3 antibody (#9661), rabbit anti-β-actin antibody (#4967), horse-radish peroxidase (HRP)-labeled anti-rabbit IgG antibody (#7074), and HRP-labeled anti-mouse IgG antibody (#7076) were purchased from Cell Signaling Technology Japan, K.K. (Tokyo, Japan). Rabbit anti-importin alpha 5 (a.k.a. KPNA1) polyclonal antibody (18137-1-AP) was purchased from Proteintech Group, Inc. (Rosemont, IL, USA). Mouse anti-LaminB1 antibody (sc-365962) was purchased from Santa Cruz Biotechnology (Santa Cruz, Dallas, TX, USA). Ambion Silencer^®^ Select Predesigned siRNA against the gene encoding ΔNp63 (#1: no. s16413, #2: no. s16411), KPNA1 (no. s223980), and Silencer^®^ Select Negative #1 Control siRNA (no. 4390843) were purchased from Thermo Fisher Scientific, Inc. (Waltham, MA, USA).

### 2.2. Cell Culture and Treatment

The human HNSCC cell lines, SAS and Ca9-22, were obtained from the RIKEN Bio-Resource Center (Tsukuba, Japan) and maintained in high-glucose Dulbecco’s modified eagle medium (DMEM; 6 Ltd., Osaka, Japan) supplemented with 10% heat-inactivated fetal bovine serum (Sigma-Aldrich) and 1% penicillin/streptomycin (P/S, Wako Pure Chemical Industries). Cells were cultured at 37 °C in a humidified atmosphere containing 5% CO_2_.

Cells (1.0 × 10^5^) were seeded in 35 mm culture dishes (Sumitomo Bakelite Co., Ltd., Tokyo, Japan) and incubated for 6 h to allow for adherence to the surface. After incubation, the cells were exposed to X-rays. After 3 days or 6 days of continued culture at 37 °C, the cells were harvested using 0.25% trypsin-ethylenediaminetetraacetic acid (Wako Pure Chemical Industries, Ltd.) and the number of viable cells was counted using a trypan blue dye exclusion assay. For the 6-day cultures, the cells were collected 3 days after irradiation and re-seeded with cells (1.0 × 10^5^). After an additional 3 days of culturing, the cells were harvested for subsequent analysis.

### 2.3. siRNA Transfection

Cells were seeded in 24-well plates (4 × 10^4^ cells; Sumitomo Bakelite Co., Ltd.) and transfected with siRNA targeting ΔNp63 using Lipofectamine^®^ RNAiMAX (Thermo Fisher Scientific, Inc.) according to the manufacturer’s protocol. Following transfection for 48 h, ΔNp63 siRNA-transfected cells were harvested for subsequent analyses. Transfection of siRNA targeting KPNA1 was performed twice, as previously reported [23]. After the second transfection, the cells were harvested and used for the analyses. The final concentration of each siRNA was 10 nM.

### 2.4. In Vitro X-ray Irradiation

Irradiation (150 kVp, 20 mA, 0.5 mm Al, and 0.3-mm Cu filters) was performed using an X-ray generator (MBR-1520R-3; Hitachi Medical Corporation, Tokyo, Japan) at 45 cm from the focus and a dose rate of 0.99–1.03 Gy/min.

### 2.5. Clonogenic Survival Assay

Clonogenic survival was determined as previously reported [24]. Briefly, the irradiated cells were harvested after culturing for 24 h and appropriate cell numbers were seeded onto 60 mm culture dishes (Sumitomo Bakelite Co., Ltd.). The cells were cultured for 8–13 days, fixed with methanol, and stained with Giemsa solution (Wako Pure Chemical Industries). The experiments were performed in triplicate. Colonies containing more than 50 cells were counted. The surviving fraction was calculated as previously described [25].

### 2.6. Apoptosis Analysis

Apoptosis analysis was conducted using FITC-annexin V and PI as previously described [23]. Annexin V^+^ cells (sum of Annexin V^+^/PI^−^ and Annexin V^+^/PI^+^ cells) were considered to be apoptotic cells.

### 2.7. SDS-PAGE and Western Blot Analysis

SDS-PAGE and Western blot analysis were performed as previously described [26]. The following primary antibodies were used: cleaved caspase-3 antibody (1:3000), α-tubulin antibody (1:3000), LaminB1 antibody (1:3000), importin alpha 5 antibody (1:3000), p63(ΔN) antibody (1:3000), and β-actin antibody (1:4000). HRP-labeled anti-rabbit IgG antibody and HRP-labeled anti-mouse IgG antibody were used as secondary antibodies (1:10,000). The antigens were visualized using Clarity^TM^ Western ECL Substrate or Clarity MAX^TM^ Western ECL Substrate (Bio-Rad Laboratories, Inc., Hercules, CA, USA). Blot stripping was performed using stripping solution (Wako Pure Chemical Industries, Ltd.). Images were captured using the Cool Saver AE-6955 (ATTO, Tokyo, Japan) or iBright 1500 Imaging System (Thermo Fisher Scientific, Inc.). Quantification of the bands was performed using iBright Analysis Software (Thermo Fisher Scientific, Inc.).

### 2.8. Subcellular Fraction

Cytoplasmic and nuclear fractions were prepared as described previously [27], with some modifications. Briefly, cells were collected using a cell lifter on ice and washed with PBS(−). The cells were then suspended in CSK buffer A (10 mM HEPES, pH 7.9, 10 mM KCl, 1.5 mM MgCl_2_, 340 mM sucrose, 0.1% Triton X-100, 10% Glycerol) containing 1 mM DTT and dissolved on ice for 10 min. After centrifugation, the supernatant was collected for cytoplasmic fractionation. The pellet was suspended in CSK buffer B (3 mM EDTA, 0.2 mM EGTA) containing 1 mM DTT and dissolved on ice for 10 min. After centrifugation, the pellet was suspended in CSK buffer B containing 1 mM DTT and dissolved on ice for 5 min. After centrifugation, the pellet (i.e., nuclear fraction) was harvested with Laemmli sample buffer containing 2-mercaptoethanol.

### 2.9. Bioinformatics and Data Analysis

Correlations between the expression of TP63 and that of each KPNA of The Cancer Genome Atlas (TCGA) HNSCC cohorts were analyzed through cBioportal for Cancer Genomics (https://www.cbioportal.org/, accessed on 24 November 2021). Data on the expression of known ΔNp63 target genes in KPNA4 knockdown Ca9-22 cells (accession number: GSE128853) were obtained from the Gene Expression Omnibus (GEO) database [7] and analyzed.

### 2.10. Statistical Analysis

Data are presented as the mean ± standard deviation (SD) of at least 3 independent experiments. Comparisons between the control and experimental groups were performed with either a two-sided Student’s *t*-test or Mann–Whitney U-test, depending on the data distribution, using Excel 2016 software (Microsoft, Washington, DC, USA) along with the add-in software Statcel 4 (The Publisher OMS Ltd., Tokyo, Japan). When the control group was designated as 1.0 (i.e., analysis of quantified data of western blotting), a one-sample t-test was performed using GraphPad Prism 9 (GraphPad Software, Inc., San Diego, CA, USA). *p* values < 0.05 were considered statistically significant.

## 3. Results

### 3.1. Effect of ΔNp63 Knockdown on Radiosensitivity and Radiation-Induced Apoptosis of HNSCC Cells

**Figure 1:**
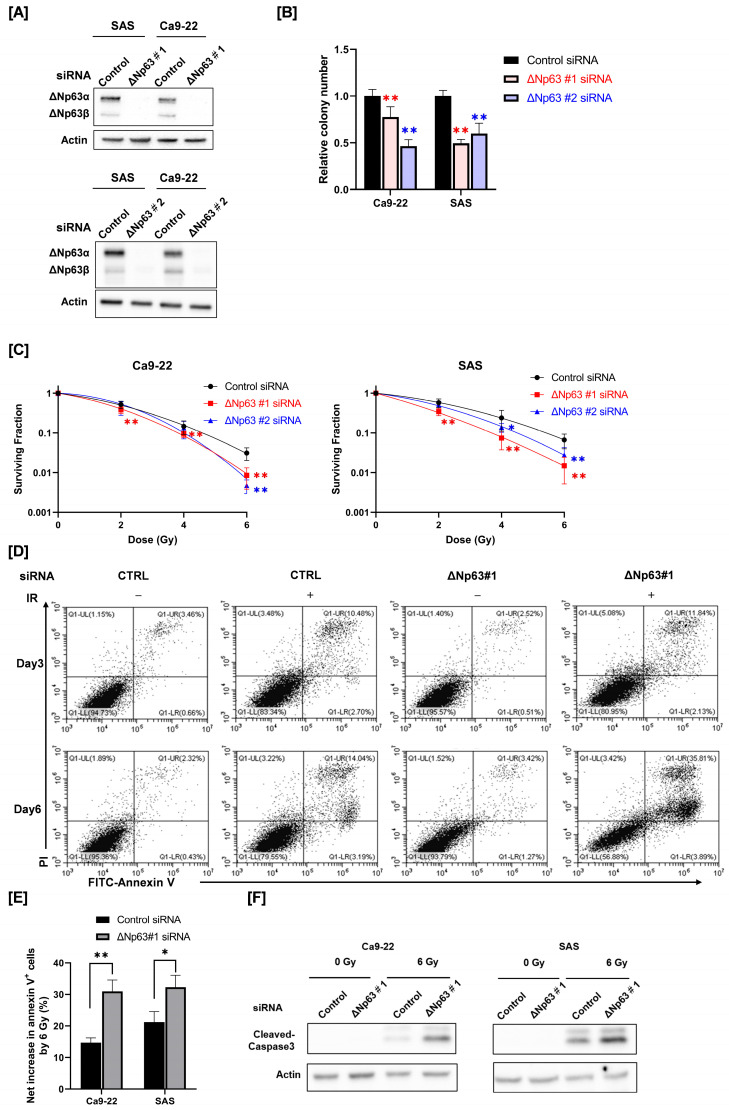
Effect of ΔNp63 knockdown on radiosensitivity and radiation-induced apoptosis of HNSCC cells. (**A**) ΔNp63 protein expression in HNSCC cells (SAS and Ca9-22) transfected with control siRNA or siRNA targeting ΔNp63 was analyzed by Western blotting. Actin was used as a loading control. (**B**) The reproductive integrity of ΔNp63 knockdown HNSCC cells was assessed using a colony formation assay. The relative colony number represents the ratio of the colony number in the treated group to that in the control group. ** *p* < 0.01 vs. control siRNA. (**C**) The radiosensitivity of ΔNp63 knockdown HNSCC cells was measured using a colony formation assay. * *p* < 0.05, ** *p* < 0.01 vs. control siRNA. (**D**–**F**) ΔNp63 knockdown HNSCC cells were prepared using ΔNp63 #1 siRNA and irradiated with X-rays. After 3 days or 6 days of culture, apoptosis was analyzed. (**D**) Representative cytograms of annexin V/PI stanning in Ca9-22 are shown. (**E**) The net increase in annexin V^+^ HNSCC cells by 6 Gy at 6 days after irradiation is shown. * *p* < 0.05, ** *p* < 0.01 vs. control siRNA. (**F**) The expression of cleaved caspase-3 proteins in 6 Gy-irradiated HNSCC cells at 6 days following irradiation was assessed by Western blot analysis. Actin was used as a loading control.

HNSCC cells (SAS and Ca9-22) were transfected with siRNA targeting ΔNp63 (#1 and #2) and ΔNp63 protein expression was analyzed by Western blotting. As shown in Figure 1A, the expression of ΔNp63α and ΔNp63β protein was detected in the control cells, whereas the transfection of siRNA targeting ΔNp63 decreased protein expression.

We evaluated the reproductive integrity of ΔNp63 knockdown HNSCC cells using a colony formation assay. As shown in Figure 1B, the reproductive integrity of ΔNp63 knockdown HNSCC cells was significantly lower compared with that of the siRNA control cells.

Next, we determined the role of ΔNp63 on the radiosensitivity of HNSCC cells. As shown in Figure 1C, the surviving fraction of irradiated HNSCC cells transfected with ΔNp63 #1 siRNA was significantly lower compared with that of the siRNA control group. Similarly, transfection with ΔNp63 #2 siRNA also decreased the surviving fraction of irradiated HNSCC cells at higher radiation doses. These results indicate that ΔNp63 contributes to the radioresistance of HNSCC cells.

We determined the role of ΔNp63 in radiation-induced apoptosis in HNSCC cells, because cellular radiosensitivity is closely associated with radiation-induced apoptosis [28,29]. As shown in Figure 1D, 6 Gy irradiation markedly increased annexin V^+^ apoptosis in Ca9-22 cells, whereas ΔNp63 knockdown alone hardly affected it. ΔNp63 knockdown resulted in a clear increase in radiation-induced apoptosis in Ca9-22 cells at day 6, but not day 3, following irradiation (Figure 1D,E). The net increase in annexin V^+^ cells by 6 Gy irradiation was estimated, and there was a significant difference between the control and the ΔNp63 knockdown HNSCC cells (Figure 1E and Supplementary Figure S1A). As is consistent with the results of apoptosis detection by annexin V^+^ staining, Western blot analysis revealed that ΔNp63 knockdown increased radiation-induced cleaved caspase-3 expression (Figure 1F and Supplementary Figure S1B), which is an execution caspase that plays a central role in apoptosis induction [30].

### 3.2. Relationship of Nuclear ΔNp63 Protein Expression and KPNA1 in Ca9-22 Cells

**Figure 2:**
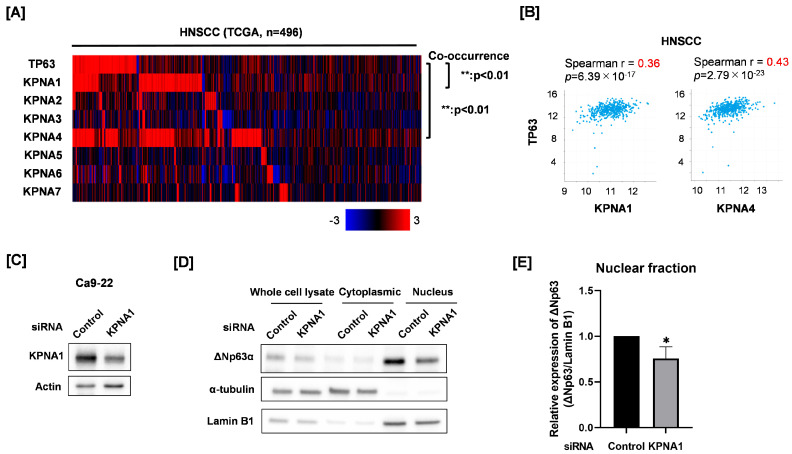
Effect of KPNA1 knockdown on nuclear expression of ΔNp63 in Ca9-22 cells. (**A**) Co-occurrence between TP63 and each KPNA mRNA expression in the TCGA HNSCC cohort was analyzed through cBioportal. (**B**) Correlation between TP63 and each of KPNA1 and KPNA4 mRNA in HNSCC from TCGA is shown. (**C**) KPNA1 protein expression in Ca9-22 cells transfected with control siRNA or siRNA against KPNA1 was analyzed by Western blotting. Actin was used as a loading control. (**D**) ΔNp63 protein expression in the cytoplasmic and nuclear fractions of KPNA1 knockdown Ca9-22 cells was analyzed by Western blotting. α-Tubulin and Lamin B1 were used as loading controls for the cytoplasmic and nuclear fractions, respectively. (**E**) Quantitation of nuclear ΔNp63 protein expression in KPNA1 knockdown Ca9-22 cells. The results are shown as relative ΔNp63 protein expression (ΔNp63/Lamin B1). * *p* < 0.05 vs. control siRNA.

We finally explored which specific KPNA molecule is involved in nuclear ΔNp63 expression. First, co-occurrence and correlation analyses were performed between TP63 mRNA and each KPNA mRNA using the TCGA HNSCC cohort. We found a significant co-occurrence and positive correlation between TP63 mRNA, KPNA1, and KPNA4 mRNA (Figure 2A,B). To investigate the relationship between ΔNp63 and KPNA4, we evaluated the effect of KPNA4 knockdown on the expression of ΔNp63 target genes by reanalyzing the gene expression data of KPNA4 knockdown Ca9-22 cells (GSE128853) [7]. We determined whether the degree of KPNA4 knockdown was associated with the expression of ΔNp63 target genes [15,31], which means that KPNA4 knockdown upregulates or downregulates ΔNp63-repressed genes or ΔNp63-activated genes, respectively. As shown in Table S1, the degree of consistency was not high (7/26), suggesting a low involvement of KPNA4 in ΔNp63 nuclear transport. Therefore, we focused on KPNA1 for this study. We prepared KPNA1 knockdown Ca9-22 cells and analyzed cytoplasmic and nuclear ΔNp63 protein expression (Figure 2C–E). As shown in Figure 2D, ΔNp63 were mainly detected in the nuclear fraction, not the cytoplasmic fraction. KPNA1 knockdown significantly decreased nuclear ΔNp63 expression of Ca9-22, whereas it hardly affected ΔNp63 expression in whole-cell lysates (Figure 2D,E). This suggests that KPNA1 is involved in nuclear ΔNp63 expression. 

## 4. Discussion

Overcoming radioresistant cancer cells is an important challenge; however, our understanding of the mechanisms of radioresistance is insufficient. Our previous study suggested that ΔNp63 is a candidate KPNB1-anchored nuclear protein that regulates the radioresistance of HNSCC cells, but it is unclear whether ΔNp63 is involved in radioresistance. Moergel et al. reported that p63 expression in oral squamous cell carcinoma tissue is associated with poor radiation response and prognosis in patients [32]. Here, our in vitro study showed that ΔNp63 knockdown increased the radiosensitivity and radiation-induced apoptosis of HNSCC cells, thus indicating that ΔNp63 is an intrinsic radioresistant factor in HNSCC cells. Since recent reports have shown that tumor cells acquire radioresistance during treatment [33], we need to investigate whether ΔNp63 knockdown can improve radioresistance in the established radioresistant HNSCC cells in a future study.

ΔNp63 is known to promote the transcription of various tumorigenesis-promoting genes that are important for SCC tumor formation [14]. Consistently with this, the results of the present study also showed that ΔNp63 knockdown decreased the reproductive integrity of HNSCC cells. Therefore, ΔNp63 may represent an effective target for overcoming not only radioresistance, but also malignant HNSCC.

Kudo et al. recently showed that ΔNp63 knockdown increases radiation-induced apoptosis in normal mammary epithelial cells expressing ΔNp63 [22]. In addition, they reported that ΔNp63α represses the p53-related radiation-induced DNA damage response, thereby inducing radioresistance of mammary epithelial stem cells [22]. Here, we found that ΔNp63 knockdown enhanced radiation-induced apoptosis in HNSCC cells. Together, these findings suggest that ΔNp63 controls resistance to radiation-induced apoptosis in both normal and cancer cells. However, because *TP53* is mutated in SAS and Ca9-22 cells [34], it is believed that ΔNp63 regulates apoptosis resistance to radiation through *TP53*-independent pathways in HNSCC.

Western blot analysis revealed that ΔNp63 knockdown facilitated radiation-induced caspase 3 activation. ΔNp63 regulates gene expression related to apoptosis. For example, Zhang et al. reported that ΔNp63 can repress the proapoptotic gene p53 upregulated modulator of apoptosis (PUMA) [35]. PUMA is one of the most potent pro-apoptotic members of the Bcl-2 family, and its expression is known to sensitize cancer cells to radiation [36,37]. Therefore, ΔNp63 knockdown may induce proapoptotic genes, such as PUMA, which results in radiosensitization. In addition, because ΔNp63 knockdown is reported to decrease the anti-apoptotic factors BCL-2 and BCL-xL [38], the regulation of anti-apoptotic factors by ΔNp63 knockdown may also be involved in radiosensitization. Since these factors are related to a mitochondria-mediated apoptotic pathway [39,40,41,42], further studies focusing on the mitochondrial changes as well as the expression of pro- and anti-apoptotic factors are needed.

Although we previously reported that KPNB1 inhibition decreased the nuclear import of ΔNp63 [10], it remains unclear which KPNA molecules are involved. We demonstrated that KPNA1 knockdown decreased nuclear ΔNp63 expression. KPNA1 is reported to carry various proteins into the nuclei, such as STAT1 and HIV-1 Vpr [43,44]; however, this is the first study showing that KPNA1 is involved in nuclear ΔNp63 expression. Because the present study only analyzed the nuclear ΔNp63 protein expression, further analyses of topics such as the interaction of KPNA1 and ΔNp63 are required in order to clarify whether KPNA1 actually imports ΔNp63 into the nuclei.

In conclusion, the present study suggests that ΔNp63 is an intrinsic radioresistance factor in HNSCC cells. Although further studies, such as in vivo experiments, are needed in order to address whether the regulation of ΔNp63 overcomes the radioresistant cells causing relapse, we believe that the strategies targeting ΔNp63, such as the degradation of ΔNp63 or the inhibition of nuclear import of ΔNp63 by drugs, may improve the effectiveness of radiation therapy for HNSCC.

## Data Availability

The data presented in this study are available within the article.

## Supplementary Materials

The following supporting information can be downloaded at: https://www.mdpi.com/article/10.3390/cimb45080394/s1, Figure S1. Effects of ΔNp63 knockdown on apoptosis induction in irradiated HNSCC cells. (A,B) ΔNp63 knockdown in HNSCC cells was conducted using ΔNp63 #2 siRNA. ΔNp63-knockdown HNSCC cells were irradiated with X-rays. After 3 days or 6 days in the culture, apoptosis was analyzed. (A) The net increase in annexin V^+^ HNSCC cells by 6 Gy at 6 days after irradiation. ** *p* < 0.01 vs. control siRNA. (B) The expression of cleaved caspase-3 proteins in 6 Gy-irradiated HNSCC cells at 6 days after irradiation was assessed by Western blot analysis. Actin was used as a loading control. Table S1. List of representative ΔNp63 target genes and the effect of KPNA4 knockdown on mRNA expression.
