# Supplementary material for: ΔNp63 Regulates Radioresistance in Human Head and Neck Squamous Carcinoma Cells
Source: Curr Issues Mol Biol. 2023 Jul 27;45(8):6262–71. doi: 10.3390/cimb45080394 (PMC10453785; doi:10.3390/cimb45080394)
Supplement: Supplementary file 1 [file cimb-45-00394-s001.zip › cimb-2504540-supplementary.pdf]

Supplementary Figure S1

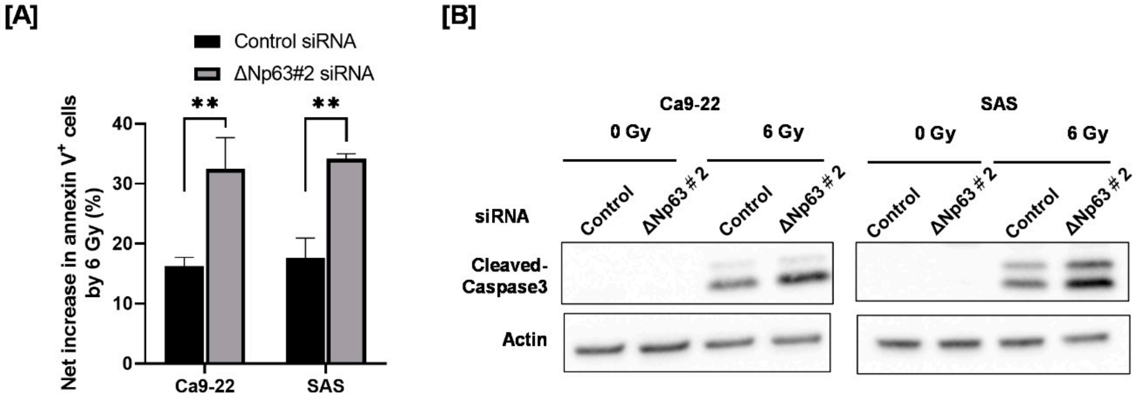

Supplementary Figure S1. Effects of ΔNp63 knockdown on apoptosis induction in irradiated HNSCC cells. (**A**, **B**) ΔNp63 knockdown in HNSCC cells was done using ΔNp63 #2 siRNA. ΔNp63 knockdown HNSCC cells were irradiated with X-rays. After 3 days or 6 days in culture, apoptosis was analyzed. (**A**) The net increase in annexin V^+ HNSCC cells by 6 Gy at 6 days after irradiation. $**p <$ 0.01 vs control siRNA. (**B**) The expression of cleaved caspase-3 proteins in 6 Gy-irradiated HNSCC cells at 6 days after irradiation was assessed by western blot analysis. Actin was used as a loading control.

Table S1. List of representative ΔNp63 target genes and the effect of KPNA4 knockdown on mRNA expression.

Gene	Effects of KPNA4 knockdown	ΔNp63 regulation	Consistency
EGFR	Down-regulation	activation	○
FGFR2	N.C.	activation	×
NRG1	Down-regulation	activation	○
IL6	Down-regulation	activation	○
CXCL8	Down-regulation	activation	○
HβDs	N.C.	activation	×
RAD18	N.C.	activation	×
BRCA2	N.C.	activation	×
Rad51	N.C.	activation	×
CYGB	N.C.	activation	×
JAG2	N.C.	activation	×
HK2	N.C.	activation	×
HAS3	N.C.	activation	×
CD44	N.C.	activation	×
ITGB4	Down-regulation	activation	○
ITGA6	N.C.	activation	×
Perp	N.C.	activation	×
ZNF750	Up-regulation	activation	×
EDAR	Up-regulation	activation	×
GLI2	N.C.	activation	×
WWC1	N.C.	repression	×
CDKN1A (p21)	N.C.	repression	×
NOTCH1	Up-regulation	repression	○
HES1	Up-regulation	repression	○
ZNF185	N.C.	repression	×
PUMA	N.C.	repression	×

Down-regulation or Up-regulation indicates that KPNA4 knockdown resulted in a 2-fold decrease or 2-fold increase, respectively. N.C. indicates unchanged. The consistency means that KPNA4 knockdown upregulates or downregulates ΔNp63-repressed genes or ΔNp63-activated genes, respectively.
